# Editorial: Robotic applications for a sustainable future

**DOI:** 10.3389/frobt.2026.1890731

**Published:** 2026-06-19

**Authors:** Franziska Kirstein, Sharath Chandra Akkaladevi, Fausto Ferreira, Amit Kumar Pandey

**Affiliations:** 1 Three Robotics, Odense, Denmark; 2 Profactor GmbH, Steyr-Gleink, Austria; 3 University of Zagreb Faculty of Electrical Engineering and Computing, Zagreb, Croatia; 4 CoE MARBLE - Centre of Excellence in Maritime Robotics and Technologies for Sustainable Blue Economy, Zagreb, Croatia; 5 Socients AI and Robotics, Paris, France

**Keywords:** circular economy, environmental monitoring, human robot collaboration, lifecycle assessment, robotic applications for sustainability, robotics, sustainability, sustainable development goals

## 1 Introduction

The global sustainability challenge is one of the most pressing Research Topic of our time. Climate change, resource depletion, biodiversity loss, and mounting waste streams demand urgent, scalable responses across all sectors of society. In this context, robotics has emerged as a powerful enabler: autonomous systems can monitor ecosystems at scale, optimize resource use in agriculture and manufacturing, and perform hazardous tasks that reduce human risk and environmental impact. Combining the physical capabilities of robots with human cognitive flexibility opens further possibilities, enabling collaborative approaches that neither humans nor robots could achieve alone.

The role of robotics in sustainability is not, however, straightforward. As robotics advances across sectors, from households to heavy industry, questions arise about whether these systems truly deliver on their sustainability promise and under what conditions. This Research Topic was motivated by the recognition that, while individual contributions to robotics for sustainability exist across many subfields, a consolidated and cross-cutting perspective was missing. We sought to capture emerging theories, technologies, and real-world applications where robotics has made a tangible difference and to foster discussion about the environmental, economic, and social dimensions of sustainability.

## 2 Overview of contributions

### 2.1 Theme 1: robotics as an enabler of environmental sustainability

#### 2.1.1 Subtheme: environmental monitoring

Drones have particularly emerged as a versatile tool for environmental monitoring across a range of applications. Bretschneider et al. provided a comprehensive analysis of the requirements, sensors, and scenarios in which a drone can be used to detect pipeline leaks, along with preliminary field results. The use of drones for environmental monitoring was also addressed by Lundquist et al., where a dedicated doctoral network focused its work on using drones to monitor wildlife both on land and at sea. Finally, Yalcinkaya et al. addressed the need for multiple robots and for better human-robot interaction in robotic fleet management, demonstrating their framework in outdoor drone operations. Indeed, given the vastness of the environment, single robots cannot efficiently cover all operational scenarios, and more sustainable solutions involving fleets of robots are required.

#### 2.1.2 Subtheme: recycling and disassembly

Building on field and infrastructure applications where robotics support environmental monitoring and operational efficiency, sustainability considerations are increasingly extending to end-of-life processes, such as recycling and disassembly. Within this subtheme, three studies collectively connected the state of the art with actionable routes to deployment. First, Ameur et al. synthesized 2 decades of research on AI-enabled robotic disassembly, structuring the landscape across optimization, perception, safety, and human–robot collaboration, and highlighting persistent gaps in datasets, standardization, and design-for-disassembly. Against this backdrop, Saenz et al. focused on automated e-waste disassembly and emphasized the importance of interoperable product and process representations for scalable deployment, supported by shared information infrastructures, such as digital twins, and emerging mechanisms, such as digital product passports, to enable transferable robot skills across heterogeneous e-waste streams. Finally, Rastegarpanah et al. addressed a bottleneck in battery circularity: the need for rapid, safe state-of-health assessments. Their industrial robotic framework automates electrochemical impedance spectroscopy using compliant manipulation and an integrated measurement stack, reducing human exposure while enabling consistent testing of battery modules. Together, these contributions reinforced the idea that sustainable disassembly requires both robust automation and the lifecycle information foundations that make circular strategies feasible at scale.

### 2.2 Theme 2: sustainable design and lifecycle thinking

The growing need for sustainable design and for considering the entire lifecycle of a product from a sustainability perspective was demonstrated by Joop van der Schoor and Göhlich through the MARBLE case study, an urban service robot for municipal litter-bin emptying. Their study showed that life cycle assessment and costing can be applied in robotics, while highlighting the limited treatment of social aspects and introducing a preliminary social assessment of the use phase. The study emphasized that sustainability is shaped not only by the robot but also by the broader service and social context.

### 2.3 Theme 3: social and systemic dimensions

Further bridging the social dimension with the systemic aspect, Fleres and Damiano proposed a perspective in sustainable robotics toward self-organizing dynamics. Using the case of Sony AIBO and its post-discontinuation ecosystem, referring to the network of owners, repair specialists, and community practices that continue after official support has ended, this study shows how repair, reuse, and stewardship can sustain robots beyond their original lifecycle. By introducing the concept of the robosphere, the article frames robots as part of evolving human–robot socio-technical ecosystems rather than as isolated products.

While considering acceptance of product reuse, McGloin et al. explored consumer attitudes toward second-hand robots for the home through a UK survey. The study found that purchase interest in second-hand robots increases significantly when a guarantee is provided, highlighting the importance of such mechanisms for reuse within a circular economy perspective. The study also identifies concerns, including cost, safety, and data security, showing that adoption depends not only on technical feasibility but also on user perceptions and purchasing conditions (e.g., take-back concepts).

## 3 Editorial observations

Across these contributions, several cross-cutting themes emerge. Sustainability in robotics consistently demands a holistic perspective, as technical performance alone is insufficient without attention to lifecycle impacts, societal and economic consequences, and the broader systems in which robots operate. Trade-offs are unavoidable: between automation and human collaboration, between operational efficiency and end-of-life responsibility, and between technological potential and its broader societal costs, including impacts on economic viability, jobs, data privacy, and equitable access across regions. While robotic applications for sustainability are increasingly well-defined and diverse, two dimensions remain largely underexplored: the sustainability of robots themselves, where lifecycle assessment is still a rare approach, and the social dimension, where methodological frameworks are still developing.

## 4 Outlook and future directions

The contributions gathered in this Research Topic suggest that robotics is indeed contributing to sustainability across a growing range of applications; however, the conditions for it to fully deliver are not yet in place. Metrics and standards for evaluating robotic sustainability are still evolving; the field would benefit from shared frameworks grounded in comparative studies that allow meaningful comparison across applications and move beyond isolated case studies. Roboticists can no longer focus solely on making robots more capable; they must also build knowledge and sensitivity toward the environmental and societal consequences of their work. While ethical considerations are increasingly present in the community, environmental sustainability remains underrepresented. As robotics takes on an ever greater role in addressing the challenges of our time, the question is no longer whether sustainability matters to the field but rather how quickly it can rise to meet that responsibility.

